# Brain and soccer: Functional patterns of brain activity during the generation of creative moves in real soccer decision‐making situations

**DOI:** 10.1002/hbm.24408

**Published:** 2018-09-26

**Authors:** Andreas Fink, Jürgen U. Bay, Karl Koschutnig, Katharina Prettenthaler, Christian Rominger, Mathias Benedek, Ilona Papousek, Elisabeth M. Weiss, Anna Seidel, Daniel Memmert

**Affiliations:** ^1^ Institute of Psychology University of Graz Graz Austria; ^2^ Institute of Exercise Training and Sport Informatics German Sport University Cologne Cologne Germany

**Keywords:** creativity, divergent thinking, domain‐specificity, functional imaging, soccer

## Abstract

This fMRI study investigated brain activity while soccer players were imagining creative moves in real soccer decision‐making situations. After presenting brief video clips of a soccer scene, participants had to imagine themselves as the acting player and think either of a creative or obvious move that might lead to a goal. Findings revealed stronger activation during trials in which the generation of obvious moves was required, relative to trials requiring creative moves. The reversed contrast (creative > obvious) showed no significant effects. Activations were mainly left‐lateralized, primarily involving the cuneus, middle temporal gyrus, and the rolandic operculum, which are known to support the processing of multimodal input from different sensory, motor and perceptual sources. Interestingly, more creative solutions in the soccer task were associated with smaller contrast values for the activation difference between obvious and creative trials, or even with more activation in the latter. Furthermore, higher trait creative potential (as assessed by a figural creativity test) was associated with stronger activation differences between both conditions. These findings suggest that with increasing soccer‐specific creative task performance, the processing of the manifold information provided by the soccer scenario becomes increasingly important, while in individuals with higher trait creative potential these processes were recruited to a minor degree. This study showed that soccer‐specific creativity tasks modulate activation levels in a network of regions supporting various cognitive functions such as semantic information processing, visual and motor imagery, and the processing and integration of sensorimotor and somatosensory information.

## 1 INTRODUCTION

Successful solutions in soccer game situations are often original and surprising, characterized by the flexible production of novel, unexpected passes, and moves (Memmert, 2017a). In making effective decisions, soccer players need to focus their attention on continuously changing conditions of the soccer scenario, to integrate task‐relevant information stored in memory, and to inhibit inappropriate solution approaches. Creative solutions in sport situations thus seem to be characterized by mechanisms that are very similar to those seen in other creativity‐related domains (for an overview see Memmert, 2015). These processes may include divergent and convergent modes of thinking (Guilford, 1967), specific attentional foci (e.g., attentional breadth; Furley, Memmert, & Heller, 2010; Kasof, 1997), domain‐specific knowledge, and associative abilities—processes that have consistently been identified as important ingredients of creativity in general (e.g., Eysenck, 1995; Runco, 2007; Sternberg & Lubart, 1996).

Several empirical research reports demonstrate that creativity is a key factor for success in soccer performance. Kempe and Memmert (2018), for example, recently investigated the level of creativity of goals scored in the FIFA World Cup 2010 and 2014, likewise in the UEFA Euro 2016. This study revealed that teams that advanced to the later rounds of the competition showed greater creativity (as assessed by soccer experts) than less successful teams. In another study, Vestberg, Gustafson, Maurex, Ingvar, and Petrovic (2012) found better performance of high division as opposed to lower division soccer players in various measures of executive functions also involving creativity related task demands (i.e., design fluency task). One crucial factor possibly contributing to higher creativity in soccer performance might be more effective attentional or visual search processes. In a study by Roca, Ford, and Memmert (2018), soccer players were required to interact with a representative life‐size video‐based simulation of attacking soccer situations. An interesting finding of this study was that more creative as compared to less creative players employed a broader attentional focus including more fixations of shorter duration and toward more informative locations of the display (as assessed by a portable eye‐movement registration system). Hence, not only the technical and physical skills but also cognitive functions of soccer players are important ingredients for successful performance (see for a review, Memmert, 2017a). Generating and implementing (including the imagination of motor executions) surprising and original solutions in a situation can be crucial for its success. In soccer this comes along with tactical creativity, which includes decision making based on observation and analysis of individual players, interaction of a player group and general team strategy (see Memmert, 2017b).

From a more general view, the domain of sports might be considered as promising field to investigate creative performance in a more ecologically valid way (cf. Lieberman, 2000; Runco & Sakamoto, 1999; Simonton, 2003). To date, the vast majority of neuroscience studies on creativity focused on (verbal) divergent thinking tasks. Available evidence allowed to identify a core creativity network underlying a broad range of divergent thinking demands (for review see Gonen‐Yaacovi et al., 2013), including regions of the lateral prefrontal cortex, which are known to support various higher order executive processes such as fluency, flexibility, or cognitive control. It moreover included a network of brain regions (i.e., left inferior parietal, superior temporal, and the inferior frontal gyri) which have been associated with semantic processes such as the activation and retrieval of internal memory representations (Binder, Desai, Graves, & Conant, 2009). A more recent meta‐analysis of fMRI studies involving open‐ended problems in musical, verbal, and visuo‐spatial domains suggests that different domains of creativity are associated with functionally specialized brain areas, supporting the idea that creativity and its neural underpinnings are specific to a particular domain (Boccia et al., 2015).

The goal of this study was to extend neuroscientific research on creativity to the domain of sports. Specifically, we investigated functional patterns of brain activity during the generation of creative solutions in soccer decision‐making situations. Each soccer scene was presented via brief video clips. Participants were asked to imagine themselves as the acting player and—depending on the respective task instruction—to think either of a creative/original (possible and promising) or an obvious/conventional move (control condition) that might lead to a goal. The general experimental test design has been validated extensively in different sport settings (basketball: Furley et al., 2010; Memmert & Furley, 2007; soccer: Memmert, Hüttermann, & Orliczek, 2013). In the domain of soccer, for instance, Memmert et al. (2013) found that better task performance was associated with more original, flexible, and adequate solutions in promotion‐oriented (focus on accomplishments and aspirations) relative to prevention‐framed (focus on safety and responsibility) soccer athletes. More recently, Fink et al. (2018) employed the soccer decision‐making task in a sample of hobby to amateur soccer players while task‐related power changes in the EEG alpha band were assessed. This study revealed that the soccer task generally elicited comparatively strong alpha power decreases (relative to a pre‐stimulus baseline) at parietal and occipital sites, indicating high visuospatial processing demands during the processing of the complex soccer scenarios. In addition, more creative task performance in the soccer task was associated with stronger alpha power desynchronization over left cortical sites, primarily involving motor related areas. This finding suggests that individuals generating more creative moves were more intensively engaged in processes related to motor or movement imagery.

The specific aim of this study was to further assess neurocognitive mechanisms associated with creative solutions in realistic soccer decision‐making situations, and to compare these findings with existing functional imaging findings on creativity (Boccia, Piccardi, Palermo, Nori, & Palmiero, 2015; Gonen‐Yaacovi et al., 2013). Along with the related EEG study (Fink et al., 2018), this functional imaging study sought to obtain a more comprehensive picture of the manifold neurocognitive mechanisms involved in the generation of creative solutions in this domain. The employed soccer decision‐making task requires participants to focus their attention on specific conditions of the soccer scenario (positions of teammates and opponents), to anticipate the behavior of other players (players emerging unexpectedly, etc.), and to think of possible passes or moves (including the motor execution) that are most promising to score a goal. The imagination of creative moves may also involve the search and retrieval of task‐relevant information stored in memory (e.g., soccer‐specific rules, technical knowledge about the execution of the pass or move, conventional task solutions, etc.). Finally, in order to generate a creative and effective move, participants are also required to evaluate the efficacy and appropriateness of the imagined move, and to inhibit inappropriate or conventional solution approaches. Creative solutions in soccer decision‐making situations may thus strongly overlap with classic divergent thinking tasks, especially with respect to the production of novel and adequate/useful ideas, imaginative mental simulation, effective memory search retrieval, and the overcoming/inhibition of typical, prevalent task solutions. Creative solutions in sport‐decision making situations seem thus on the one hand recruit rather domain‐general brain networks supporting executive functions and semantic memory demands (cf. Gonen‐Yaacovi et al., 2013). On the other hand, relevant creativity literature also clearly indicates that creativity and its neural underpinnings are specific to a particular domain (Baer, 1998; Boccia et al., 2015). For instance, studies in the visual creativity domain (e.g., Aziz‐Zadeh, Liew, & Dandekar, 2013; Pidgeon et al., 2016; Rominger et al., 2018) provide consistent evidence of the involvement of brain networks supporting visuospatial processes and motor related imagery. In a similar vein, in investigating brain activity during musical improvisation in jazz pianists, Limb and Braun (2008) found among others a widespread activation of brain regions supporting sensorimotor functions. They suggested that this finding might not necessarily reflect an increase in motor activity related to the playing of the instrument, but also processes related to the encoding and implementation of novel motor sequences that are implicated in spontaneous musical improvisation. On the basis of these findings, we expect that functionally more specialized networks that support movement related imagery may likewise play a crucial role in generating creative solutions in soccer decision‐making situations (cf. Fink et al., 2018).

## 2 METHODS

### 2.1 Participants

Thirty men in the age range between 18 and 32 years (*M* = 23.90; *SD* = 3.44) participated in this study. As important inclusion criteria, participants were required to have been actively playing soccer for at least 10 years (at least once per week). On average, participants have been actively playing soccer for approximately 17 years (*M* = 17.43, *SD* = 3.85), and they indicated to play soccer for about 7 hrs per week (*M* = 6.78, *SD* = 4.15). All participants were right‐handed (as assessed by the hand dominance test, HDT; Steingrüber & Lienert, 1971; Papousek & Schulter, 1999), non‐medicated, and written informed consent was obtained. The study was approved by the local ethics committee.

### 2.2 Experimental task during FMRI assessment

**Figure 1:**
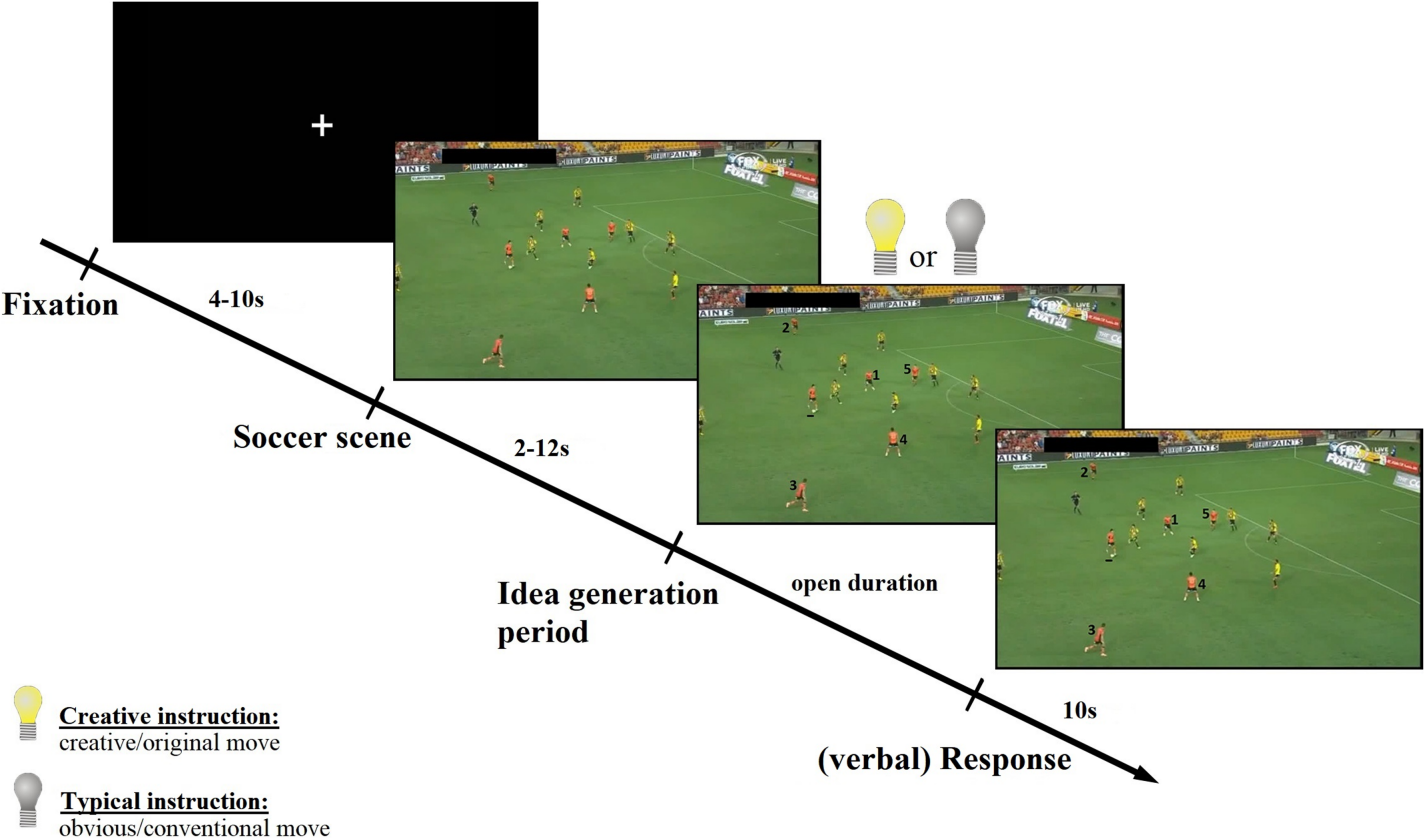
Schematic time course of a trial of the soccer decision‐making task during fMRI assessment. A trial started with the presentation of a fixation cross for 10 s. Afterwards, brief video clips of real soccer decision‐making situations were shown (ranging from 2 to 12 s). During the idea generation period a fixed image of the soccer scene remained visible on the screen, signaling participants to imagine themselves as the acting player, and—depending on the respective task instruction—to think either of an obvious/conventional (switched off bulb, control condition) or a creative/original move (lightened bulb) to score a goal. When they thought of a solution/move they were instructed to press the IDEA button, and to vocalize the imagined move (max 10 s; e.g., pass to 1, then pass to 3, etc.) [Color figure can be viewed at http://wileyonlinelibrary.com]

Participants worked on a modified version of the standardized video task in soccer (SVT‐S), in which participants were required to mentally generate moves with the intention to score a goal in a given soccer decision‐making situation. The objectivity, reliability and validity of the SVT‐S has been established in previous studies (Memmert, 2013; Memmert et al., 2013). The stimuli for the SVT‐S were videos of the German and Australian soccer league. It is important to note that in comparison with the original version of the SVT‐S (e.g., Memmert et al., 2013), the employed soccer task had to be modified to a considerable amount in order to be reasonable realizable during fMRI assessment, especially with respect to the specific instructions that were given to the participants (generating either obvious or creative moves), the specific kind and exact duration of stimulus presentation, and the mode of responding (only one solution per trial). As shown in Figure 1, each trial started with the presentation of a fixation cross for a time period of 10 s. Afterwards, brief video clips of real soccer decision‐making situations were shown (ranging from 2 to 12 s in length). The fixed image of the soccer scene marked the beginning of the idea generation period, in which participants had to imagine themselves as the acting player of the attacking team (who was marked by an underline in the fixed image) and—depending on the respective task instruction—to think either of an obvious/conventional (control condition) or a creative/original move to score a goal. This assignment was not made randomly but with regard to the originality range of solutions a single video clip showed. Video clips tending to show more original solutions were assigned to the creative condition. Video clips tending to show more solutions with a rating at the unoriginal end of the scale were assigned to the standard condition (quantification of originality see below). The respective task condition was indicated by a lightened (i.e., creative/original) or switched off (i.e., obvious/conventional) bulb, respectively (see Figure 1). During the idea generation period, the fixed image of the soccer scene remained visible on the screen and the players of the attacking team were assigned with numbers to make them clearly identifiable. Participants were not allowed to speak during the idea generation period. They were instructed to press the IDEA button with the dominant right hand as soon as they thought of a solution/move, and then (during the response period, 10 s) to vocalize the imagined move in shorthand notes (e.g., pass to 1, then pass to 3, hitting a cross to 5, and then header shot by 5, etc.). The oral responses were recorded via a microphone and transcribed for further analysis. In each condition (creative/original and control condition) 15 items were presented, resulting in a total number of 30 trials. The presentation of trials was randomized and the total fMRI session took about 15–20 min.

### 2.3 Quantification of task performance

The 30 videos administered during fMRI assessment were taken from a pool of videos of previous studies of our laboratory (see e.g., Memmert et al., 2013). In those studies, the videos were shown to a group of participants who had to find as many solutions that would lead to a goal as possible. Afterwards, the answers were classified based on the first pass participants imagined to execute. Up to four soccer experts with the highest soccer qualification (UEFA A license) viewed the answers, along with the respective video, and rated the originality of each answer category on scales from 1 to 5 (1 = not original, 5 = very original; Memmert et al., 2013) or 1 to 7 (1 = not original at all, 7 = very original). The inter‐judge reliability coefficient was above the critical limit of .80 (intraclass correlation coefficient). The raters were asked to keep in mind that the main aim was to score a goal, when rating the answers.

The available answer categories for the soccer scenes were used for quantifying soccer task performance in this study. For this purpose, the answer of every participant to every video was assigned to the suitable answer category by the authors. Most of these responses were consistent with the rated categories established in previous studies (see e.g., Memmert et al., 2013). In seven out of 30 videos one or more answers did not fit into any of the available categories. For this reason, another soccer expert (UEFA A license) rated these specific answers' originality, along with seeing the respective video clip, on a scale from 1 to 5 or 1 to 7. The average of the expert ratings for the respective answer category was the originality score a participant received for his/her answer in this category. Afterwards, these scores were divided by the stimulus‐specific maximum (5 or 7, yielding a creativity score per stimulus/soccer scene ranging from 0 to 1) and averaged across the trials of the control and the creative condition, respectively, to obtain a creative performance score for each participant in both conditions.

### 2.4 Assessment of general creative potential

In order to assess the influence of trait creative potential on creative solutions in the soccer task, a figural creativity test (“Test zum Schöpferischen Denken – Zeichnerisch,” TSD‐Z; Urban & Jellen, 1995) was administered. This test requires participants to complete abstract picture fragments (printed on a test sheet) in a free‐associative, original way. The time limit is 15 min. According to the instructions given in the test manual, the generated drawings were evaluated with respect to 14 different criteria (e.g., unconventionality, inclusion of new elements, graphic combinations, etc.), resulting in a total score for the creative potential of the participants.

### 2.5 FMRI data acquisition

Imaging was performed on a 3T MRI scanner MAGNETOM Skyra (Siemens Medical Systems, Erlangen, Germany) using a 32‐channel head coil. Structural images were acquired using a MPRAGE T1‐weighted sequence (TR = 1,680 ms, TE = 1.89 ms, inversion time = 1,000 ms, flip angle = 8°, 192 sagittal slices, FOV = 224 × 224 mm, distance factor = 50%, slice thickness = 0.88 mm). BOLD‐sensitive T2*‐weighted functional images were acquired using a single shot gradient‐echo EPI pulse sequence (TR = 2,520 ms, TE = 30 ms, flip angle = 90°, slice thickness = 3.3 mm, 10% distance factor, matrix size = 66 × 66, FoV = 218 mm, 38 axial slices per volume, order descending). Head motion was restricted using firm padding that surrounded the head. To record the verbal responses of the participants, an MR compatible microphone was used (FOMRI‐III, Optoacoustics Ltd., Moshav Mazor, Israel). Stimuli were presented using the Software Presentation (Neurobehavioral Systems, Albany, CA).

### 2.6 FMRI data analyses

Functional MRI data analysis was performed using SPM 12 software (v6906; Wellcome Department of Imaging Neuroscience, London, UK), which ran in a MATLAB 2015b environment (MathWorks Inc., Natick, MA). The Data Processing Assistant for Resting‐State fMRI (DPARSF, v4.1) (Yan, Wang, Zuo, & Zang, 2016) was used for preprocessing. Images were slice‐time and motion corrected. Each individual structural scan was then co‐registered with the mean function image and then segmented into GM, WM, and CSF. The DARTEL‐approach was used to create a group‐specific template for a more accurate normalization. Resulting flow fields were then applied to bring the preprocessed function images into the MNI‐space (3 mm isotropic voxels). Finally, functional images were smoothed with a Gaussian kernel of 9 mm. Effects were estimated based on the GLM implemented in SPM12 including the experimental conditions “Obvious Moves” and “Creative Moves.” Verbal response, video clips, and rest period were also entered in the model as regressors of no interest. The subject‐specific estimates of the contrast between the creative and the control condition were computed and then entered into a one‐sample *t* test treating subject as a random effect. A voxel‐wise FWE correction (at *p* < .05) was used for corrections for multiple comparisons. Only activation clusters exceeding a spatial extent threshold of 10 voxels (3 × 3 × 3 mm) are reported.

## 3 RESULTS

### 3.1 Brain activation during the generation of obvious versus creative moves

**Figure 2:**
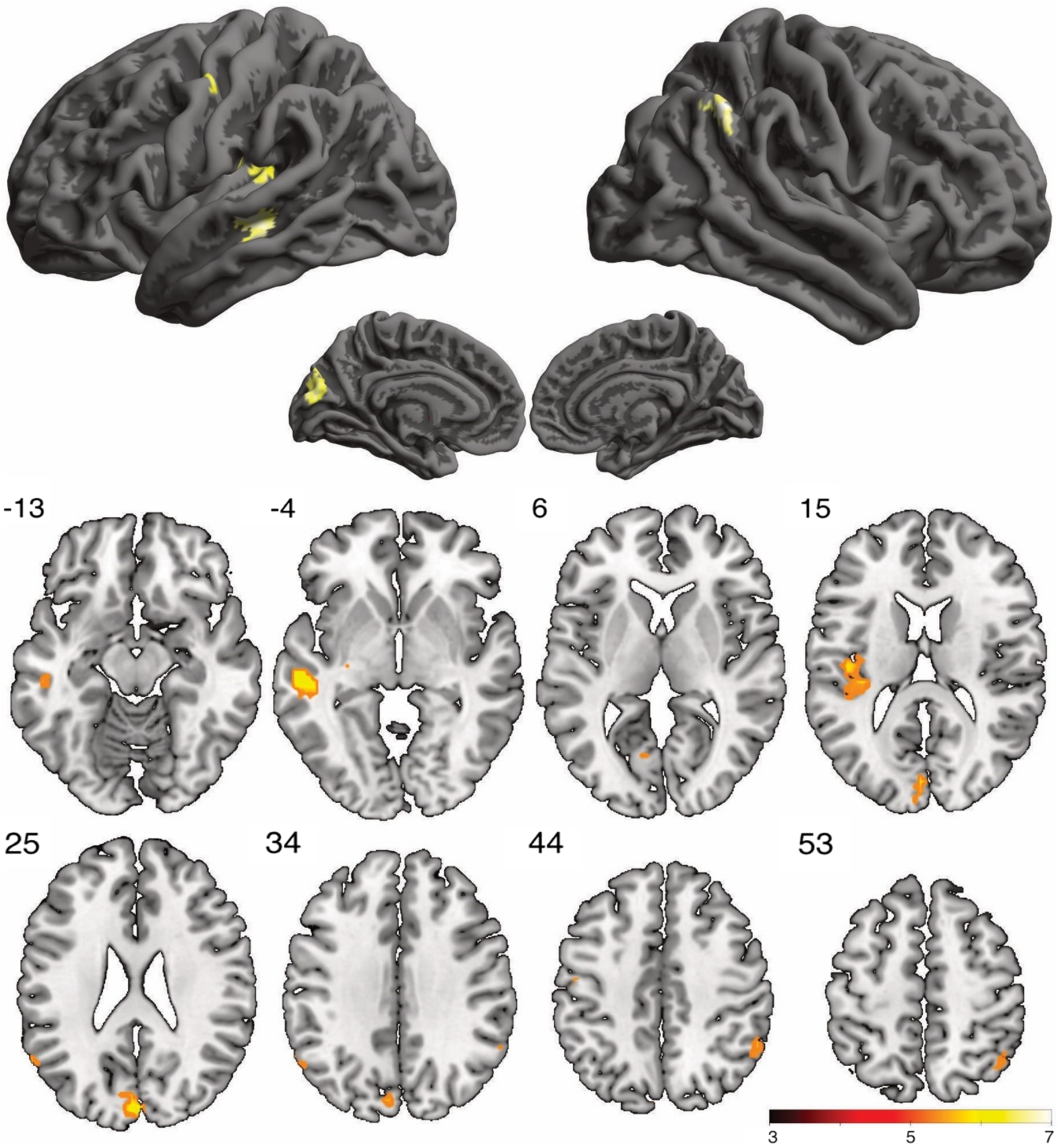
Significantly activated clusters during the generation of obvious > creative moves in soccer decision‐making situations (voxel‐wise FWE corrected at *p* < .05) [Color figure can be viewed at http://wileyonlinelibrary.com]

Thinking of obvious task solutions (control condition), as compared to imagining creative/original moves in soccer decision‐making situations, resulted in stronger activation in a mainly left‐lateralized network including the cuneus, middle temporal gyrus, the rolandic operculum, and smaller clusters involving the left angular and postcentral gyrus, and the left pallidum (see Table 1, Figure 2), along with stronger activation in the right inferior parietal cortex involving the angular gyrus. There were no significant activation clusters with more activation in the creative versus control condition.

**Table 1:** Significant activation clusters during the generation of obvious > creative moves in soccer decision‐making situations (voxel‐wise FWE corrected at *p* < .05, only cluster >10 voxel are reported)

Cluster	MNI (Peak)	*k*	*t*	Brain region (AAL)
1	−3 −90 21	103	6.61	Cuneus L
2	−57 −24 0	92	6.32	Temporal mid L, temporal sup L
3	−36 −27 15	55	6.01	Rolandic operculum L, temporal sup L
4	54 –54 39	29	6.11	Parietal inf R, angular R
5	−60 −60 27	23	6.37	Angular L
6	42 –60 57	12	5.87	Parietal inf R, angular R
7	−48 −15 51	10	5.67	Postcentral L
8	−24 −9 0	10	5.93	Pallidum L

MNI = Montreal Neurological Institute; AAL = Automated Anatomical Labeling; L = left hemisphere; R = right hemisphere; inf = inferior; mid = middle; sup = superior.

### 3.2 Correlations with originality in the soccer task and trait creative potential

**Figure 3:**
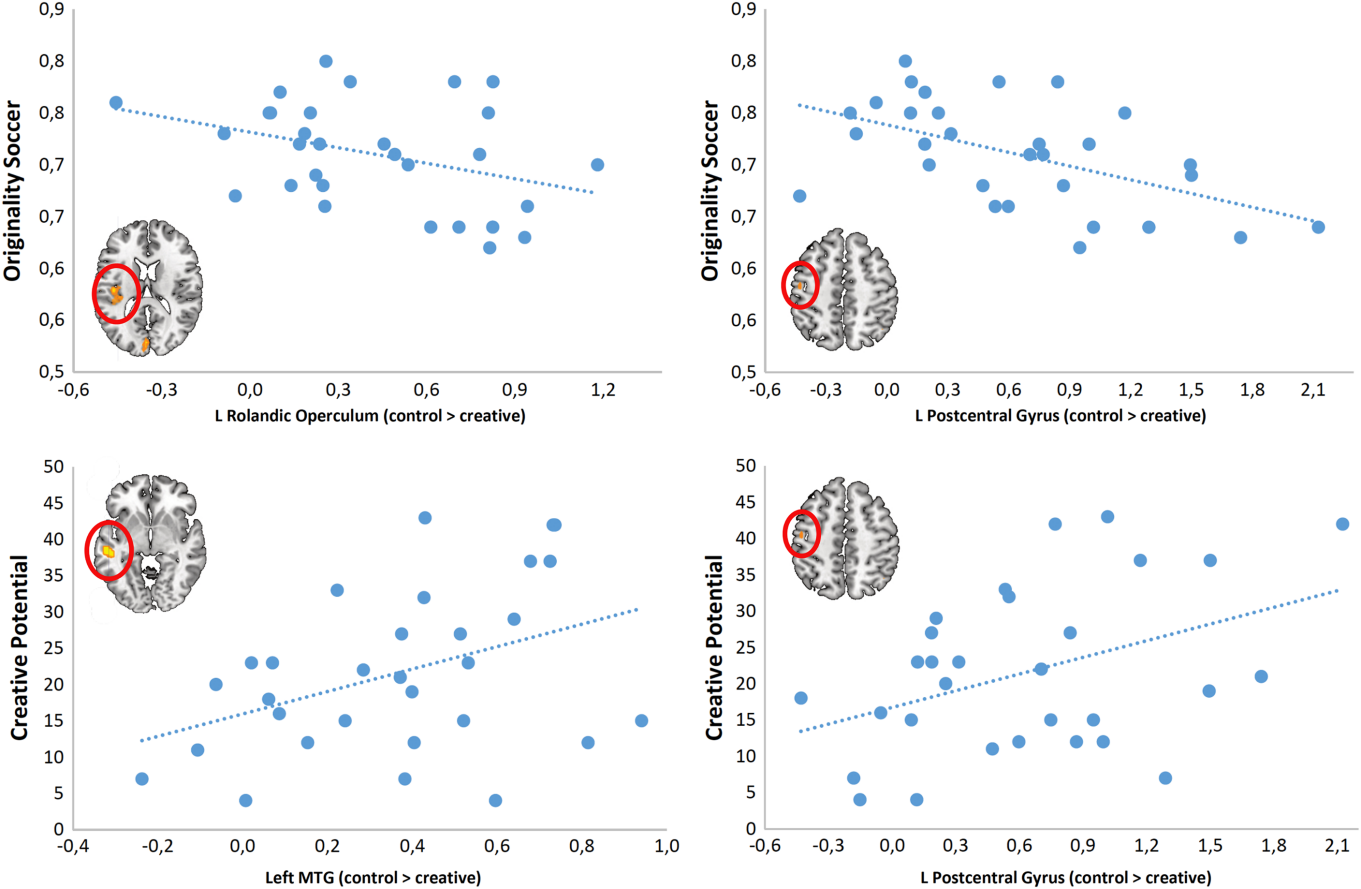
Originality in the creative condition of the soccer task was significantly negatively correlated with the estimates of the control > creative contrast in the left rolandic operculum and in the left postcentral gyrus, indicating less pronounced (or even a reversed pattern of) activation differences between both conditions with increasing originality in the soccer task. In contrast, trait creative potential was positively correlated with the contrast estimates in the left middle temporal gyrus and in the left postcentral gyrus [Color figure can be viewed at http://wileyonlinelibrary.com]

In order to test potential associations among task performance (originality) in the soccer task and the identified patterns of brain activity during performance of this task, contrast estimates were computed for the significant activation clusters in the control > creative contrast (see Table 1) and correlated with the originality scores of the soccer task. Extraction of these functionally defined Regions of Interest (ROI) was performed using the SPM 12 REX toolbox (Whitfield‐Gabrieli, 2009). As shown in Table 2, originality in the creative condition of the soccer task displayed a consistent negative pattern of correlations with the functionally defined ROIs, reaching statistical significance (*p* < .05) for the left rolandic operculum and the left postcentral gyrus, and trends toward significance (*p* < .10) for the left cuneus and the right parietal cortex (see also Figure 3). This suggests that with increasing originality in the soccer task the activation difference between the control and the creative condition becomes increasingly less pronounced, or even slightly reversed (see Figure 3).

**Table 2:** Correlations of contrast estimates (control > creative) with the originality measure in the creative condition of the soccer task and creative potential (TSD‐Z)

	Originality soccer	Creative potential (TSD‐Z)
Cuneus L	−.35+	.13
Temporal mid L	−.23	.41*
Rolandic operculum L	−.37*	.29
Parietal inf R	−.33+	−.12
Angular L	−.28	.15
Parietal inf R	−.28	.09
Postcentral L	−.53**	.41*
Pallidum L	−.18	.34+

** *p* < .01, *p < .05, ^+^ *p* < .10.

Interestingly, the observed pattern of correlations for creative potential (as assessed by a figural creativity test) was clearly different from the domain‐specific creativity measure obtained during performance of the soccer task. Creative potential showed mostly positive associations with the contrast estimates (control > creative), which were significant (*p* < .05) for the left middle temporal gyrus and the left postcentral gyrus—very close to those regions for which significant negative correlations with soccer‐specific originality were found (Table 2, Figure 3). This indicates that the higher the creative potential of an individual, the stronger is the activation of the control relative to the creative condition (or the lower the activation of the creative relative to the control condition, respectively).

### 3.3 Correlations with expertise in soccer

To assess some facets of participants' expertise in soccer, participants were asked to indicate the current amount of soccer training per week and the highest soccer league they have ever been playing in their career. The amount of training per week ranged from 0 to 12 hr (*M* = 4.35; *SD* = 2.83). The majority of participants indicated that they were actively playing in soccer clubs, the highest soccer league the participants indicated they had ever played ranged up to the third‐highest national league; only four participants were not actively playing in soccer clubs.

As shown in Table 3, both indicators of soccer‐specific expertise showed a pattern of mostly negative correlations with the identified brain network, similar to the pattern of correlations with the originality measure of the soccer task. Specifically, the amount of training per week was associated with less pronounced activation differences (control > creative) in the left cuneus and the right inferior parietal cortex, and in the left middle temporal gyrus for the highest league participants indicated that they were actively playing (see Table 3).

**Table 3:** Correlations of contrast estimates (control > creative) with indicators of soccer‐specific expertise

	Training in hours/week	Highest league
Cuneus L	−.44*	−.24
Temporal mid L	−.28	−.40*
Rolandic operculum L	.02	−.06
Parietal inf R	−.39*	−.22
Angular L	.04	−.21
Parietal inf R	−.25	−.27
Postcentral L	−.13	−.20
Pallidum L	.11	−.02

* *p* < .05; Spearman rho was used to assess the correlation between brain activation and the highest league participants indicated that they were ever playing.

### 3.4 Behavioral results

The creative condition of the soccer task resulted in more original responses (*M* = 0.71, *SD* = 0.05) than the control condition (*M* = 0.61, *SD* = 0.04; *t*(29) = 8.48, *p* < .001). The mean reaction time in the control condition (*M* = 7.38 s, *SD* = 2.84) was significantly shorter than in the creative condition (*M* = 10.00s, *SD* = 3.99; *t*(29) = 6.59, *p* < .001).

There was no significant correlation between the originality scores in the control and in the creative condition (*r* = .03, *p* = .88), and none of the task performance scores was significantly associated with trait creative potential (TSD‐Z; control condition: *r* = .01, *p* = .96: creative condition: *r* = −.09, *p* = .63). Likewise, there were no significant correlations of soccer‐specific expertise with soccer task performance in the control and creative condition and with creative potential (*r* ranging between −.11 and .12).

## 4 DISCUSSION

The generation of obvious as compared to creative moves in real‐life soccer decision‐making situations revealed activations in a mainly left‐lateralized network including the cuneus, middle temporal gyrus, the rolandic operculum, along with smaller activation clusters involving the left angular and postcentral gyrus, and the left pallidum. Overall, this network of brain regions supports various higher‐order cognitive functions such as semantic information processing (Binder et al., 2009), visual and motor imagery (Kosslyn, Ganis, & Thompson, 2001; Szameitat, McNamara, Shen, & Sterr, 2012), likewise the processing and integration of sensorimotor and somatosensory information (Eickhoff et al., 2010). Especially the cluster involving the rolandic operculum appears to overlap with a cortical network that is thought to be implicated in self‐referential processes involving self‐location in space (Ventre‐Dominey, 2014), by integrating visual, vestibular, and somatosensory information to “generate a multi‐modal neuronal representation of subject motion and orientation in space” (Karnath, 2001, p.572; see also Eickhoff, Weiss, Amunts, Fink, & Zilles, 2006; Eickhoff et al., 2010; Lopez, Blanke, & Mast, 2012). These processes might be especially relevant during the processing of the complex real‐life soccer scene. In a quite similar vein, the middle temporal gyrus is thought to have heteromodal characteristics (Binder et al., 2009), that is to act as a high level convergence zone (Binder & Desai, 2011), receiving and integrating multimodal input from different sensory, motor and perceptual sources, this way facilitating the efficient storage and retrieval of complex concepts and event knowledge (Binder & Desai, 2011). In addition, this study revealed stronger activation in the control versus creative condition in the right inferior parietal cortex involving the angular gyrus. This network is known to have a particular role in attention mechanisms and spatial cognition (Seghier, 2013), supporting the processing of salient new events (Singh‐Curry & Husain, 2009), or the automatic allocation of attention to task‐relevant information (Ciaramelli, Grady, & Moscovitch, 2008; see also Cabeza, Ciaramelli, Olson, & Moscovitch, 2008). Seghier (2013), moreover, specifically highlighted the particular role of the angular gyrus in integrating spatial information with conceptual knowledge, which might be relevant in processing complex real‐life soccer scenarios. It therefore seems that the generation of obvious as compared to creative moves in soccer decision‐making situations relied more strongly on the processing of the manifold stimulus characteristics provided by the soccer scenario, while those same processes were recruited to a minor degree in imagining creative/original moves.

Strikingly, more creative performance in the creative condition of the soccer decision‐making task was consistently associated with less pronounced activation differences between the control and the creative condition, or even a slightly reversed pattern of activation differences. This negative relation was significant for the left rolandic operculum and the left postcentral gyrus, and at a trend level for the left cuneus and the right parietal cortex (see Figure 3). A quite similar pattern of findings was observed for the indicators of soccer‐specific expertise (amount of training per week, highest soccer league), which were likewise negatively associated with the contrast estimates, especially in the left cuneus, the left middle temporal gyrus and the right inferior parietal cortex (see Table 3). Considering the prominent role of this network in the processing and integration of semantic, visual, spatial, sensorimotor and somatosensory information (e.g., Binder et al., 2009; Binder & Desai, 2011; Eickhoff et al., 2006, 2010; Karnath, 2001; Lopez et al., 2012; Seghier, 2013), a possible interpretation of this finding could be that both in individuals showing more creative soccer task performance and in individuals with higher soccer‐specific expertise the recruitment of these processes becomes increasingly important. Interestingly, there were no significant correlations between soccer‐specific expertise and originality in the soccer task. That creativity and expertise must not necessarily be associated is not a surprising finding. In a study with chess masters, Bilalić, McLeod, and Gobet (2008) found that “ordinary” chess experts (three SDs above average) were more prone to inflexibility of thought induced by prior knowledge (choosing well‐known solutions), while the “super” experts (five SDs above average) looked for and found better task solutions. In future research it will thus be exciting to see how performance of elite soccer players relates to different facets of creativity.

The findings of this study also add evidence to the common notion that different domains of creativity are organized in functionally specialized brain networks, depending on the respective creativity domain. While in verbal divergent thinking demands a predominantly left‐lateralized brain network mostly involving the inferior frontal gyrus and the inferior parietal cortex has been identified (e.g., Benedek et al., 2014; Fink et al., 2009, 2010; Kleibeuker, Koolschijn, Jolles, De Dreu, & Crone, 2013), other types of creative behavior such as visual creativity, which involve more similar task demands as the creative soccer task employed in this study, also recruit motor related brain networks, indicating that processes such as motor imagery are likewise important components of creativity (Aziz‐Zadeh et al., 2013; Boccia et al., 2015). A similar finding has been observed during musical improvisation in jazz pianists (Limb & Braun, 2008). Likewise, this study suggested that with increasing creative soccer task performance the processing of sensorimotor and somatosensory information becomes increasingly important—quite similar to the result pattern obtained in the related EEG study (Fink et al., 2018), which revealed that more creative soccer task was associated with activation at motor related cortical sites.

Another important finding of this study was that participants' creative potential, as assessed by a psychometric creativity test, was also significantly correlated with functional patterns of brain activity during performance of the soccer task, though, when compared to the soccer‐specific creativity measure, diametrically in the opposite way. Higher creative potential was associated with stronger activation differences between both conditions in the left middle temporal gyrus and in the left postcentral gyrus, that is the higher the creative potential of the participants the higher is the activation of the control relative to the creative condition (or the lower the activation of the creative condition relative to control, respectively). This finding adds further evidence to the common view of domain‐specificity of creativity and nicely complements the result pattern found in the related EEG study by Fink et al. (2018). This study revealed that the creative potential of the individuals was globally positively associated with alpha power at all cortical sites, while the soccer‐specific creativity score was associated with rather specific effects at motor related sites. Increases in alpha power during creative ideation have been interpreted as reflecting high internal processing demands, characterized by the shielding of ongoing cognitive processes from potentially interfering, task‐irrelevant information, thereby supporting processes such as effective memory retrieval, imaginative thought processes and mental simulation (Fink & Benedek, 2014). The positive association of alpha power during performance of the soccer task and creative potential might thus indicate more internally driven thought processes that are less strongly concerned with specific stimulus‐driven bottom‐up processing demands. At the same time, this fMRI study revealed that higher creative potential was associated with stronger relative deactivation of the creative versus control condition in brain regions supporting the integration and processing of visual, perceptual, sensorimotor and somatosensory information, probably indicating that individuals with higher creative potential solved the creative relative to the control condition in a less externally oriented or less stimulus‐driven manner. However, a direct comparison of the EEG (Fink et al., 2018) and the current fMRI study is somewhat complicated by the fact that the EEG alpha power changes during soccer task performance relate to a pre‐stimulus reference (resting) period, while the current fMRI study focused on the contrast estimates between the (active) control and creative condition. Nevertheless, the findings of Fink et al. (2018) and this study clearly indicate that creativity and its neural signatures are specific to a particular domain (Baer, 1998). This supports the common notion that creativity is not a uniform process, but rather involves manifold neurocognitive processes depending on the respective creativity domain.

What still remains somewhat puzzling in the overall pattern of findings is the fact that the results of the task contrast seem to be at odds with the findings obtained in the correlational analyses. On the one hand, the creative condition of the soccer task generally (i.e., across all participants) yielded less brain activation than the obvious condition in a brain network supporting visual and motor imagery, and the processing of sensorimotor and somatosensory information (Figure 2). However, at the individual level on the other hand, the activation difference between both conditions diminished, or even reversed, as creativity in the soccer task increased (Figure 3). It seems that when participants are instructed to respond creatively (i.e., thinking of new, original but still effective ways to score a goal) they generally adopt a task strategy characterized by more internal mental simulation (operating on relevant memory content) and less bottom‐up processing of specific characteristics of the soccer scene. Such a thinking style seems to be especially pronounced in participants with higher creative potential, as indicated by the positive pattern of correlations with the contrast estimates in this study. While this strategy is generally effective in producing more creative solutions compared to trials asking for conventional responses, higher creativity within the creative condition of the soccer task was actually achieved with more bottom‐up processing of the soccer scene (similar to the common condition, but likely focusing on different aspects of the scene).

This pattern of findings is strikingly similar to that observed in the accompanying EEG study (Fink et al., 2018), which revealed more alpha activity (indicating more internally driven thought processes) over parieto‐occipital sites during thinking of creative versus obvious solutions, along with a global positive association between alpha power and creative potential. But again, higher creativity in the soccer task was linked to brain activity over more task‐specific cortical sites. Consistent with the above line of reasoning, the EEG findings were interpreted to reflect that more creative task performance is linked to higher processing of specific (visual, motor‐related) information provided by the soccer scene. Taken together, our findings hence suggest that the overall instruction to respond creatively versus conventionally in the soccer task yields a different pattern of neurocognitive processes than that associated with inter‐individual variations in soccer‐specific creativity within the creative condition of the task. It seems that different facets of creativity are operating here: The task instruction to think creatively (vs. conventionally) characterized by the recruitment of more internally oriented thought processes, and soccer‐specific creativity within the creative task condition being more strongly linked to the processing of stimulus‐related information. These findings point to an interesting dissociation between domain‐general creative potential and soccer‐specific creativity as they exhibit different modulations of brain activity in a soccer decision‐making task.

While this study took a fresh and novel step forward in studying creative processes in a more ecologically valid way, there are also some important limitations. First, since the idea generation periods were self‐paced, the analysis intervals differed with respect to their length, both inter‐ and intra‐individually. Especially the fact that the creative condition was significantly longer than the control condition could possibly also influence the pattern of results. However, various control analyses with fixed time intervals (e.g., 2 or 5 s after stimulus onset) revealed no single brain region that was more activated in the creative versus control condition (even at the uncorrected level). Rather, they revealed stronger activation in the control condition in brain regions similar to the findings reported here, though, however, the significance of these analyses is limited given the considerably lower number of time segments and trials available for analysis (resulting in lower reliability). A further limitation of the self‐paced response mode could be also seen in the fact that there might be various individual differences in the strategies participants are pursuing in generating a creative move. Hence, an analysis of brain activity in shorter and fixed time intervals, which also considers different stages of the creative ideation process, and which may resemble real‐play conditions more realistically (e.g., time pressure, etc.), would be extremely valuable here. Also, in this study participants were explicitly instructed to think either of an obvious or a creative move. It would be also interesting to see the “typical” behavior of participants in such real‐life soccer decision‐making situations and how this is reflected in brain activity. Notwithstanding these restrictions we conclude that the applied experimental paradigm facilitates the investigation of creative behavior in a novel, ecologically valid way. This study revealed that finding creative soccer moves is a complex cognitive process involving the processing of multimodal input from different sensory, motor and perceptual sources, along with domain‐specific mechanisms such as visual or motor related mental imagination.

## References

[hbm24408-bib-0001] Aziz‐Zadeh, L. , Liew, S.‐L. , & Dandekar, F. (2013). Exploring the neural correlates of visual creativity. Social Cognitive and Affective Neuroscience, 8, 475–480. 10.1093/scan/nss021 22349801 PMC3624959

[hbm24408-bib-0002] Baer, J. (1998). The case for domain specificity of creativity. Creativity Research Journal, 11, 173–177. 10.1207/s15326934crj1102_7

[hbm24408-bib-0003] Benedek, M. , Jauk, E. , Fink, A. , Koschutnig, K. , Reishofer, G. , Ebner, G. , & Neubauer, A. C. (2014). To create or to recall? Neural mechanisms underlying the generation of creative new ideas. NeuroImage, 88, 125–133. 10.1016/j.neuroimage.2013.11.021 24269573 PMC3991848

[hbm24408-bib-0005] Bilalić, M. , McLeod, P. , & Gobet, F. (2008). Inflexibility of experts—Reality or myth? Quantifying the Einstellung effect in chess masters. Cognitive Psychology, 56, 73–102. 10.1016/j.cogpsych.2007.02.001 17418112

[hbm24408-bib-0006] Binder, J. R. , & Desai, R. H. (2011). The neurobiology of semantic memory. Trends in Cognitive Sciences, 15, 527–536. 10.1016/j.tics.2011.10.001 22001867 PMC3350748

[hbm24408-bib-0007] Binder, J. R. , Desai, R. H. , Graves, W. W. , & Conant, L. L. (2009). Where is the semantic system? A critical review and meta‐analysis of 120 functional neuroimaging studies. Cerebral Cortex, 19, 2767–2796. 10.1093/cercor/bhp055 19329570 PMC2774390

[hbm24408-bib-0008] Boccia, M. , Piccardi, L. , Palermo, L. , Nori, R. , & Palmiero, M. (2015). Where do bright ideas occur in our brain? Meta‐analytic evidence from neuroimaging studies of domain‐specific creativity. Frontiers in Psychology, 6, 1195 10.3389/fpsyg.2015.01195 26322002 PMC4531218

[hbm24408-bib-0009] Cabeza, R. , Ciaramelli, E. , Olson, I. R. , & Moscovitch, M. (2008). Parietal cortex and episodic memory: An attentional account. Nature Reviews Neuroscience, 9, 613–625. 10.1038/nrn2459 18641668 PMC2692883

[hbm24408-bib-0010] Ciaramelli, E. , Grady, C. L. , & Moscovitch, M. (2008). Top‐down and bottom‐up attention to memory: A hypothesis (AtoM) on the role of the posterior parietal cortex in memory retrieval. Neuropsychologia, 46, 1828–1851. 10.1016/j.neuropsychologia.2008.03.022 18471837

[hbm24408-bib-0011] Eickhoff, S. B. , Jbabdi, S. , Caspers, S. , Laird, A. R. , Fox, P. T. , Zilles, K. , & Behrens, T. E. (2010). Anatomical and functional connectivity of cytoarchitectonic areas within the human parietal operculum. The Journal of Neuroscience, 30, 6409–6421. 10.1523/jneurosci.5664-09.2010 20445067 PMC4791040

[hbm24408-bib-0012] Eickhoff, S. B. , Weiss, P. H. , Amunts, K. , Fink, G. R. , & Zilles, K. (2006). Identifying human parieto‐insular vestibular cortex using fMRI and cytoarchitectonic mapping. Human Brain Mapping, 27, 611–621. 10.1002/hbm.20205 16281284 PMC6871353

[hbm24408-bib-0013] Eysenck, H. J. (1995). Genius: The natural history of creativity. Cambridge [i.a.]: Cambridge University Press.

[hbm24408-bib-0052] Fink, A. , & Benedek, M. (2014). EEG alpha power and creative ideation. Neuroscience and Biobehavioral Reviews, 44, 111–123. 10.1016/j.neubiorev.2012.12.002 23246442 PMC4020761

[hbm24408-bib-0014] Fink, A. , Grabner, R. H. , Benedek, M. , Reishofer, G. , Hauswirth, V. , Fally, M. , … Neubauer, A. C. (2009). The creative brain: Investigation of brain activity during creative problem solving by means of EEG and fMRI. Human Brain Mapping, 30, 734–748. 10.1002/hbm.20538 18266217 PMC6871103

[hbm24408-bib-0015] Fink, A. , Grabner, R. H. , Gebauer, D. , Reishofer, G. , Koschutnig, K. , & Ebner, F. (2010). Enhancing creativity by means of cognitive stimulation: Evidence from an fMRI study. NeuroImage, 52, 1687–1695. 10.1016/j.neuroimage.2010.05.072 20561898

[hbm24408-bib-0016] Fink, A. , Rominger, C. , Benedek, M. , Perchtold, C. , Papousek, I. , Weiss, E. M. , … Memmert, D. (2018). EEG alpha activity during imagining creative moves in soccer decision‐making situations. Neuropsychologia, 114, 118–124. 10.1016/j.neuropsychologia.2018.04.025 29702162

[hbm24408-bib-0017] Furley, P. , Memmert, D. , & Heller, C. (2010). The dark side of visual awareness in sport—Inattentional blindness in a real‐world basketball task. Attention, Perception, & Psychophysics, 72, 1327–1337. 10.3758/APP.72.5.1327 20601714

[hbm24408-bib-0018] Gonen‐Yaacovi, G. , de Souza, L. C. , Levy, R. , Urbanski, M. , Josse, G. , & Volle, E. (2013). Rostral and caudal prefrontal contribution to creativity: A meta‐analysis of functional imaging data. Frontiers in Human Neuroscience, 7, 465 10.3389/fnhum.2013.00465 23966927 PMC3743130

[hbm24408-bib-0019] Guilford, J. P. (1967). The nature of human intelligence. New York, NY: McGraw Hill.

[hbm24408-bib-0020] Karnath, H. O. (2001). New insights into the functions of the superior temporal cortex. Nature Reviews Neuroscience, 2, 568–576. 10.1038/35086057 11484000

[hbm24408-bib-0021] Kasof, J. (1997). Creativity and breadth of attention. Creativity Research Journal, 10, 303–315. 10.1207/s15326934crj1004_2

[hbm24408-bib-0022] Kempe, M. , & Memmert, D. (2018). “Good, better, creative”: The influence of creativity on goal scoring in elite soccer. Journal of Sports Sciences, 36, 2419–2423. 10.1080/02640414.2018.1459153 29623764

[hbm24408-bib-0023] Kleibeuker, S. W. , Koolschijn, P. C. M. P. , Jolles, D. D. , De Dreu, C. K. W. , & Crone, E. A. (2013). The neural coding of creative idea generation across adolescence and early adulthood. Frontiers in Human Neuroscience, 7, 905 10.3389/fnhum.2013.00905. 24416008 PMC3874541

[hbm24408-bib-0024] Kosslyn, S. M. , Ganis, G. , & Thompson, W. L. (2001). Neural foundations of imagery. Nature Reviews Neuroscience, 2, 635–642. 10.1038/35090055 11533731

[hbm24408-bib-0025] Lieberman, M. D. (2000). Intuition: A social cognitive neuroscience approach. Psychological Bulletin, 126, 109–137. 10.1037//0033-2909.126.1.109 10668352

[hbm24408-bib-0026] Limb, C. J. , & Braun, A. R. (2008). Neural substrates of spontaneous musical performance: An fMRI study of jazz improvisation. PLoS One, 3(2), e1679 10.1371/journal.pone.0001679 18301756 PMC2244806

[hbm24408-bib-0027] Lopez, C. , Blanke, O. , & Mast, F. W. (2012). The human vestibular cortex revealed by coordinate‐based activation likelihood estimation meta‐analysis. Neuroscience, 212, 159–179. 10.1016/j.neuroscience.2012.03.028 22516007

[hbm24408-bib-0028] Memmert, D. (2013). Tactical creativity In McGarry T., O'Donoghue P., & Sampaio J. (Eds.), Routledge handbook of sports performance analysis (pp. 297–308). Abingdon, England: Routledge.

[hbm24408-bib-0029] Memmert, D. (2015). Teaching tactical creativity in sport: Research and practice. Abingdon, England: Routledge.

[hbm24408-bib-0030] Memmert, D. (2017a). Tactical creativity in sport In Kaufman J., Glăveanu V., & Baer J. (Eds.), The Cambridge handbook of creativity across domains (pp. 479–491). Cambridge, England: Cambridge University Press 10.1017/9781316274385.026

[hbm24408-bib-0031] Memmert, D. (2017b). Sports and creativity In Runco M. A. & Pritzker S. R. (Eds.), Encyclopedia of creativity (2nd ed., pp. 373–378). San Diego, CA: Academic Press.

[hbm24408-bib-0032] Memmert, D. , & Furley, P. (2007). “I Spy With My Little Eye!”: Breadth of attention, inattentional blindness, and tactical decision making in team sports. Journal of Sport & Exercise Psychology, 29, 365–347. 10.1123/jsep.29.3.365 17876972

[hbm24408-bib-0033] Memmert, D. , Hüttermann, S. , & Orliczek, J. (2013). Decide like Lionel Messi! The impact of regulatory focus on divergent thinking in sports. Journal of Applied Social Psychology, 43, 2163–2167. 10.1111/jasp.12159

[hbm24408-bib-0034] Papousek, I. , & Schulter, G. (1999). Quantitative assessment of five behavioural laterality measures: Distributions of scores and intercorrelations among right‐handers. Laterality, 4, 345–362. 10.1080/713754344 15513122

[hbm24408-bib-0035] Pidgeon, L. M. , Grealy, M. , Duffy, A. H. B. , Hay, L. , McTeague, C. , Vuletic, T. , … Gilbert, S. J. (2016). Functional neuroimaging of visual creativity: A systematic review and meta‐analysis. Brain and Behavior, 6, 1–26e00540. 10.1002/brb3.540 PMC5064346 27781148

[hbm24408-bib-0036] Roca, A. , Ford, P. R. , & Memmert, D. (2018). Creative decision making and visual search behavior in skilled soccer players. PLoS One, 13(7), e0199381 10.1371/journal.pone.0199381 29990320 PMC6039007

[hbm24408-bib-0037] Rominger, C. , Papousek, I. , Perchtold, C.M. , Weber, B. , Weiss, E.M. , & Fink, A. (2018). The creative brain in the figural domain: Distinct patterns of EEG alpha power during idea generation and idea elaboration. Neuropsychologia. [published online ahead of print] doi: 10.1016/j.neuropsychologia.2018.02.013 29452125

[hbm24408-bib-0039] Runco, M. A. (2007). Creativity – Theories and themes: Research, development, and practice. Burlington, MA: Elsevier Academic Press.

[hbm24408-bib-0040] Runco, M. A. , & Sakamoto, S. O. (1999). Experimental studies of creativity In Sternberg R. J. (Ed.), Handbook of creativity (pp. 62–92). Cambridge, England: Cambridge University Press.

[hbm24408-bib-0041] Seghier, M. L. (2013). The angular gyrus: Multiple functions and multiple subdivisions. The Neuroscientist, 19, 43–61. 10.1177/1073858412440596 22547530 PMC4107834

[hbm24408-bib-0042] Simonton, D. K. (2003). Scientific creativity as constrained stochastic behavior: The integration of product, person and process. Psychological Bulletin, 129, 475–494. 10.1037/0033-2909.129.4.475 12848217

[hbm24408-bib-0043] Singh‐Curry, V. , & Husain, M. (2009). The functional role of the inferior parietal lobe in the dorsal and ventral stream dichotomy. Neuropsychologia, 47, 1434–1448. 10.1016/j.neuropsychologia.2008.11.033 19138694 PMC2697316

[hbm24408-bib-0044] Steingrüber, H.‐J. , & Lienert, G. (1971). Hand‐Dominanz‐test: H‐D‐T; Handanweisung. Göttingen, Germany: Hogrefe.

[hbm24408-bib-0045] Sternberg, R. J. , & Lubart, T. I. (1996). Investing in creativity. American Psychologist, 51, 677–688. 10.1037/0003-066X.51.7.677

[hbm24408-bib-0046] Szameitat, A. J. , McNamara, A. , Shen, S. , & Sterr, A. (2012). Neural activation and functional connectivity during motor imagery of bimanual everyday actions. PLoS One, 7(6), e38506 10.1371/journal.pone.0038506 22701655 PMC3368848

[hbm24408-bib-0047] Urban, K. K. , & Jellen, H. G. (1995). *TSD‐Z: Test zum Schöpferischen Denken ‐ Zeichnerisch (manual). TCT‐DP Test for Creative Thinking‐Drawing Production (manual)*. Frankfurt, Germany: Swets Test Services.

[hbm24408-bib-0048] Ventre‐Dominey, J. (2014). Vestibular function in the temporal and parietal cortex: Distinct velocity and inertial processing pathways. Frontiers in Integrative Neuroscience, 8, 53 10.3389/fnint.2014.00053 25071481 PMC4082317

[hbm24408-bib-0049] Vestberg, T. , Gustafson, R. , Maurex, L. , Ingvar, M. , & Petrovic, P. (2012). Executive functions predict the success of top‐soccer players. PLoS One, 7(4), e34731 10.1371/journal.pone.0034731 22496850 PMC3319604

[hbm24408-bib-0050] Whitfield‐Gabrieli, S. (2009). Region of interest extraction (REX) toolbox. Boston, MA: Elsevier.

[hbm24408-bib-0051] Yan, C. G. , Wang, X. D. , Zuo, X. N. , & Zang, Y. F. (2016). DPABI: Data Processing & Analysis for (resting‐state) brain imaging. Neuroinformatics, 14, 339–351. 10.1007/s12021-016-9299-4 27075850

